# A Review of Remote Sensing Image Dehazing

**DOI:** 10.3390/s21113926

**Published:** 2021-06-07

**Authors:** Juping Liu, Shiju Wang, Xin Wang, Mingye Ju, Dengyin Zhang

**Affiliations:** 1 School of Internet of Things, Nanjing University of Posts and Telecommunications, Nanjing 210000, China; Q19010312@njupt.edu.cn (J.L.); 1020072120@njupt.edu.cn (S.W.); 1220076503@njupt.edu.cn (X.W.); zhangdy@njupt.edu.cn (D.Z.); 2 School of Business, Macquarie University, Sydney 2109, Australia

**Keywords:** remote sensing image, image dehazing, retinex, dark channel, dehazenet

## Abstract

Remote sensing (RS) is one of the data collection technologies that help explore more earth surface information. However, RS data captured by satellite are susceptible to particles suspended during the imaging process, especially for data with visible light band. To make up for such deficiency, numerous dehazing work and efforts have been made recently, whose strategy is to directly restore single hazy data without the need for using any extra information. In this paper, we first classify the current available algorithm into three categories, i.e., image enhancement, physical dehazing, and data-driven. The advantages and disadvantages of each type of algorithm are then summarized in detail. Finally, the evaluation indicators used to rank the recovery performance and the application scenario of the RS data haze removal technique are discussed, respectively. In addition, some common deficiencies of current available methods and future research focus are elaborated.

## 1. Introduction

Remote sensing (RS) was widely used in military affairs [1], e.g., missile early warning [2], military reconnaissance [3], and surveying [4]. With the popularity of satellites, it is also being used for civilian purposes increasingly, such as land planning and crop yield surveys [5]. Despite its usefulness, RS images or data taken by satellites are easy to be affected by the fog or haze during the imaging process, which makes images low contrast or dim color [6] and decreases the performance of computer vision tasks such as object detection [7]. This adverse effect not only reduces the visual quality of RS images, but also limits such precious RS data from being effectively applied.

To collect high-quality RS data, the most intuitive way is to perform imaging under good visibility and ideal illumination [8]. However, in some practical applications [9], it is urgent to shoot the location of the incident in time and continuously. Once haze or fog fills the atmosphere, RS imaging would lose its original worth. Therefore, a robust and real-time haze removal algorithm is very critical for restoring the RS data.

Singh et al. [10] summarized the image dehazing algorithms from several perspectives including: Theory, mathematical models, and performance measures. He divided dehazing algorithms into seven categories, i.e., depth estimation, wavelet, enhancement, filtering, supervised learning, fusion and meta-heuristic techniques, and introduced the strengths and weaknesses, respectively. Although the content of Ref. [10] is very comprehensive, its explanation of some related algorithms is not detailed enough. Unlike Ref. [10], this paper would group the current RS image dehazing algorithms into three categories. The first one is based on image enhancement, the main advantage of which is having a low complexity to ensure real-time performance. However, it does not work well for most situations due to the ignored imaging theory. The second one is physical dehazing [11], which is to impose hand craft prior knowledge on the atmospheric scattering model (ASM) to estimate the imaging parameters. Regrettably, the existing prior knowledge cannot be satisfied to all the scenes, thereby hindering the practicality of this type of algorithm. Benefiting from a sharp development of deep-learning [12,13], the last one utilizes convolutional neural network (CNN) [14], generative adversarial networks (GAN) [15] or other networks to train an end-to-end dehazing system [16]. Although high-quality results can be restored by these created networks, the recovery performance depends too much on the sample dataset used.

The rest of the paper is organized as follows. Section 2 describes the haze imaging model and gives the deriving of its formulas. Image enhancement such as Histogram Equalization and Retinex are discussed in Section 3. Section 4 mainly describes two well-known physical dehazing, i.e., dark channel prior and haze-lines prior dehazing. Since data-driven methods have a strong learning ability, some data-driven dehazing techniques such as Dehazenet, MSCNN, and AOD-NET, are discussed in Section 5. Section 6 describes the different quality metrics used to evaluate the recovery performance of different techniques. In Section 7, the significance and application of RS dehazing techniques to society are discussed. Future work is elaborated in Section 8. The concluding remarks are presented in Section 9. 

## 2. Haze Degradation Model

### 2.1. Causes of Image Degradation in Hazy Weather

Haze is a common natural phenomenon in the real-world, which is formed by suspended particles, e.g., aerosols, dust, compounds, and tiny water droplets in the atmosphere [17]. These suspended particles, on the one hand, would interfere with the propagation of scene reflected light during the imaging procedure, i.e., causing scattering and refraction [18]. On the other hand, the atmospheric light will also participate in this imaging process, which becomes more serious as the depth increases. Therefore, these two factors lead to the massive loss of the reflected light of target object, and are bound to result in blurred texture, excessive brightness, decreased contrast, and dim color [19].

### 2.2. Atmospheric Scattering Model

According to the above analysis, Nayer and Narasimhan [19,20,21,22] consider that the scene reflected light in hazy atmosphere can be separated into two parts: Attenuation and Airlight.

**Figure 1:**
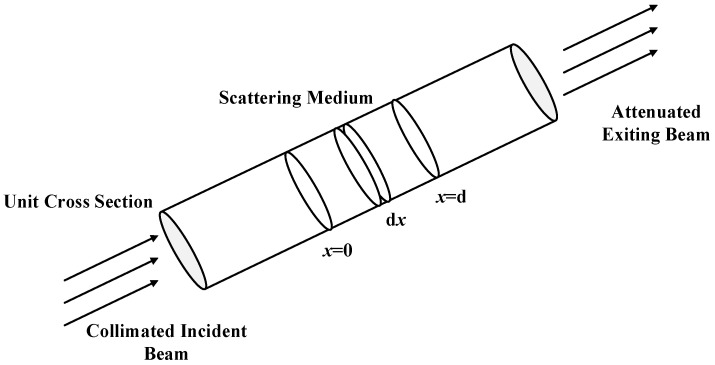
Attenuation of a collimated beam of light by suspended particles.

Attenuation refers to the scene reflected light that reaches the observer after being scattered by atmospheric particles, and this procedure is shown in Figure 1. As can be seen from this figure, when the incident light (or reflected light) enters the scattering medium, the intensity changes for each distance dx is:

(1) dE(x,λ)=−β(λ)·E(x,λ)dx,

where β(λ) is the scattering coefficient used to measure the ability of a medium to scatter light at different wavelengths, and λ is the wavelength of light. To calculate definite integrals on both sides of the above formula within the range of x∈[0, d], the following equation is given as:

(2) $$E\left(x,\lambda \right)=E_{0}\left(\lambda \right)\cdot e^{-\int_{0}^{d}\beta \left(\lambda \right)dx},$$

where $E_{0}\;\left(\lambda \right)$ represents the radiance at x=0. Assuming that each point on the scene can be regarded as a light source, the flux of light per unit area is inversely proportional to the square of the distance, which yields:

(3) $$E\left(d,\lambda \right)=\frac{L_{h}\left(\infty ,\lambda \right)\cdot \rho \cdot e^{-\beta \left(\lambda \right)d}}{d^{2}},$$

where $L_{h}\left(\infty ,\lambda \right)$ stands for the atmospheric light at infinity, and  ρ represents the ability of an object to reflect light.

**Figure 2:**
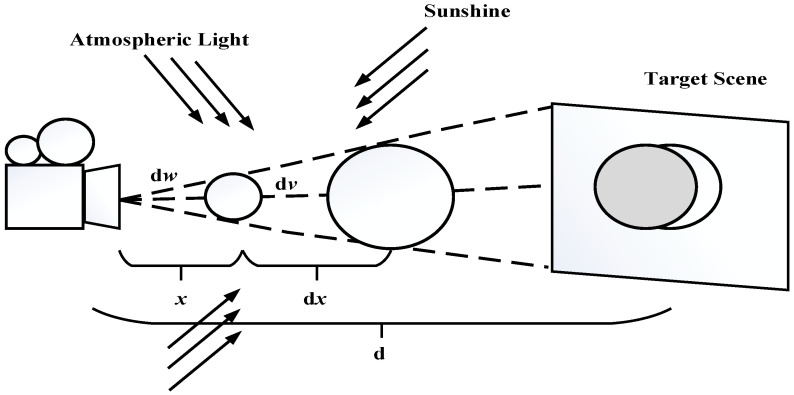
The atmosphere scatters environmental illumination in the direction of the observer.

Airlight represents the component of atmospheric light involved in the imaging process, which is depicted in Figure 2. Assuming that the imaging ranges are the same and the angle between the tangential light and the horizontal light is dω, thus we can produce its luminous intensity:

(4) $$dI\left(x,\lambda \right)=dV\cdot k\cdot \beta \left(\lambda \right)=d\omega \cdot x^{2}dx\cdot k\cdot \beta \left(\lambda \right),$$

where $dV=d\omega *x^{2}*dx$ is the volume and k is a constant. If dV is regarded as a light source with brightness d(x,λ), the scattered light intensity can be further expressed as:

(5) $$dE\left(x,\;\lambda \right)=\frac{dI\left(x,\lambda \right)\cdot e^{-\beta \left(\lambda \right)x}}{x^{2}},$$

From the combination Equations (4) and (5) and dL(x,λ)=(dE(x,λ))/dω, we have

(6) $$L\left(d,\lambda \right)=k\left(1-e^{-\beta \left(\lambda \right)d}\right),$$

Now, extending the atmospheric scattering model to RGB space:

(7) $$E\left(d\right)=\frac{L_{h}\left(\infty ,\lambda \right)\cdot \rho \cdot e^{-\beta \left(\lambda \right)d}}{d^{2}}\cdot \tilde{D}+L_{h}\left(\infty ,\lambda \right)\cdot \left(1-e^{-\beta \left(\lambda \right)d}\right)\cdot \tilde{A},$$

where $\tilde{D}$ and $\tilde{A}$ are the unit direction vectors of scene pixels and atmospheric color in RGB space, respectively. Therefore, in the RGB space, ASM can be modeled as:

(8) $$I\left(x\right)=\frac{L_{\infty }\left(\lambda \right)\cdot \rho \left(x\right)}{d^{2}}\cdot e^{-\beta \left(\lambda \right)dx}+L_{\infty }\left(\lambda \right)\cdot \left(1-e^{-\beta \left(\lambda \right)dx}\right)=J\left(x\right)\cdot t\left(x\right)+\left(1-t\left(x\right)\right)\cdot A,$$

where x is the pixel coordinates, I is the observed intensity, J is the true radiance of the scene point imaged at x, A is the global atmospheric light, and t is the medium transmission. In ASM, the first term on the right side, named Direct Attenuation, is used to describe the direct impact of scene reflection light caused by haze, which usually attenuates exponentially with the scene depth d. The second term is called airight, which increases with the scene depth d [23].

## 3. Dehazing Using Image Enhancement

Image enhancement based dehazing does not consider the physical model of image degradation but improves the image quality by increasing the contrast of an image [24]. In these algorithms, the most representative is histogram equalization, Retinex algorithm, and homomorphic filtering.

### 3.1. Histogram Equalization

Histogram equalization [25] is a classic image enhancement method. Mathematically, it can be detailed by:

(9) $$s_{k}=T\left(r_{k}\right)=\left(L-1\right)\cdot \sum_{j=0}^{k}p_{r}\left(r_{j}\right)=\frac{\left(L-1\right)}{MN}\cdot \sum_{j=0}^{k}n_{j},\;k=0,1,2,\ldots ,L-1,$$

where M and N are the height and width of an image, $n_{j}$ is the total number of pixels in the image with grayscale j, L is the total number of grayscale levels in the image (8-bit image corresponding to 256), and r and s represent the pixel grayscale before and after histogram equalization, respectively. MN is the total number of pixels in the image, and $p_{r}\left(r_{j}\right)$ is the probability of occurrence of grayscale j and $p_{r}\left(r_{j}\right)=\frac{n_{j}}{MN}$. The main advantage of histogram equalization is low computational cost and easy to implement [26]. Therefore, it has the potential to deal with RS data with a high resolution. However, it only works well on an image with heavy haze due to its powerful overall contrast enhancement ability. To address this issue, Kim et al. [27,28] proposed a local histogram equalization, which can be divided into three strategies: Sub-block non-overlapped, fully overlapped sub-block, and partially overlapped sub-block. Although they can produce a visual haze-free result for most cases, the recovery color appears to be darker than the real one. In fact, due to the same scene depth in RS data, these images usually have a uniform haze distribution, thus histogram equalization is more suitable for the RS image.

### 3.2. Retinex

Retinex theory was found by Edwin Land et al. [29] in 1963, which is a combination of retina and cortex and simulates the imaging process of the human eye. Based on this fact, it is also called a cerebral cortex theory. 

#### 3.2.1. Retinex Algorithm

Retinex algorithm holds that the image observed by the eye can be represented by the product of the reflection and irradiation component:

(10) $$I_{i}\left(x,y\right)=R_{i}\left(x,y\right)\cdot L_{i}\left(x,y\right),$$

where i∈{R,G,B} represent the three color bands, $I_{i}\left(x,y\right)$ represents the actual observed value, $R_{i}\left(x,y\right)$ represents the reflection component, and $L_{i}\left(x,y\right)$ represents the irradiation component. $R_{i}\left(x,y\right)$ can be obtained by calculating the irradiation component from the original image. For the convenience of calculation, Equation (10) is transformed into a logarithm form, i.e.,

(11) $$log\left[R_{i}\left(x,y\right)\right]=log\left[I_{i}\left(x,y\right)\right]-log\left[L_{i}\left(x,y\right)\right],$$

#### 3.2.2. Single Scale Retinex

Jobson [30] proposed the Single Scale Retinex algorithm. It can estimate the irradiation component by weighting the average of the pixels in the neighborhood, which is expressed as follows:

(12) $$L_{i}\left(x,y\right)=I_{i}\left(x,y\right)*G\left(x,y\right),$$

where ∗ is the convolution operation, and G(x,y) is the Gaussian function, which can be described by:

(13) $$G\left(x,y\right)=\frac{1}{\sqrt{2\pi }}e^{-\frac{\left(x^{2}+y^{2}\right)}{2\sigma^{2}}},$$

where ∬G(x,y)dxdy=1, σ is the radius range. When the value of σ is small, more details will be displayed, but color distortion may occur. On the contrary, when the value of σ is large, the color information in the image is more natural, while the details are easy to lose. Combining Equations (12) and (13), it can be expressed as follows:

(14) $$log\left[R_{i}\left(x,y\right)\right]=log\left[I_{i}\left(x,y\right)\right]-log\left[I_{i}\left(x,y\right)*G\left(x,y\right)\right],$$

Here, we remark that the SSR algorithm only uses a single scale to estimate the unknown parameter, thus it may significantly reduce the enhancement quality [31].

#### 3.2.3. Multi-Scale Retinex

To overcome the above flaw, MSR [32] is designed to weigh the average values of different reflection components, and it is calculated as follows:

(15) $$logR_{i}\left(x,y\right)=\sum_{k=1}^{N}\omega_{k}\left\{logI_{i}\left(x,y\right)-log\left[I_{i}\left(x,y\right)*G_{k}\left(x,y\right)\right]\right\},$$

where N represents the number of scales, $G_{k}\left(x,y\right)$ represents the k-th Gaussian function, and $\omega_{k}$ is the weight of the k-th scale, satisfying $\sum_{k=1}^{N}\omega_{k}=1$. If N=1, the MSR is transformed into SSR. Although MSR has the ability to make up for the shortcomings of SSR, it still produces the halo effect and the overall luminance is insufficient.

#### 3.2.4. Multi-Scale Retinex with Color Restoration

Since the MSR algorithm processes the three RGB channels separately, the change of color ratio will inevitably lead to color distortion. Therefore, Rahman et al. [33] and Jobson et al. [34,35] proposed MSRCR to adjust the reflection component R(x,y) by introducing a color restoration factor, that is:

(16) $$R_{MSRCR_{i}}\left(x,y\right)=C_{i}\left(x,y\right)\cdot R_{MSR_{i}}\left(x,y\right)\;\;\;\;i\epsilon \left\{R,G,B\right\},$$

(17) $$C_{i}\left(x,y\right)=log\left[\frac{\alpha I_{i}\left(x,y\right)}{\sum_{n=1}^{N}I_{n}\left(x,y\right)}\right],$$

where $C_{i}$ is the color restoration factor of the i-th channel, and α is a non-linear adjustment factor. In general, MSRCR can have a stronger robustness and restore richer detailed information than MSR. However, the complexity of the algorithm is increased undoubtedly.

### 3.3. Other Dehazing Algorithms Based on Image Enhancement

Homomorphic filtering [36] is one of the well-known image enhancement methods and is based on the frequency domain of irradiation-reflection. In this method, the irradiation component is used to determine the image’s grayscale variation, mainly corresponding to the low-frequency information. Moreover, the reflection component determines the image’s edge details, mainly corresponding to the high-frequency information. The homomorphic filtering method aims to use a certain filter function to reduce the low-frequency information and increase the high-frequency information [37]. This means that the homomorphic filtering method and the Retinex algorithm are very similar in the calculation [38]. Both of them divide the image into two parts: The irradiation component and the reflection. However, the difference is that the former processes the image in the frequency domain, and the latter is in the space domain. Homomorphic filtering is able to remove the shadows caused by uneven illumination, and can maintain the original information of the image. However, it needs two Fourier transforms, which take up a larger computing space. The basic idea of wavelet transform is similar to the above homomorphic filtering. Different frequency features of the original hazy image are obtained by the wavelet transform. It can enhance the image’s detailed information to achieve the dehazing image [39], but it cannot apply to a situation where the image is too bright or dark. Ancuti et al. [40] applied a white balance and a contrast enhancing procedure to enhance the visibility of hazy images. However, it has not been shown to be physically valid.

### 3.4. Remote Sensing Image Dehazing Based on Image Enhancement

Shi et al. [41] developed an image enhancement algorithm to restore hazy RS images by combining the Retinex algorithm and chromaticity ratio. It introduces the color information of the original image when using the Retinex algorithm, and also overcomes the color distortion easily caused by the histogram equalization and the grayish image caused by the Retinex algorithm. S. Huang et al. [42] proposed a dehazing algorithm called the new Urban Remote Sensing Haze Removal (URSHR) algorithm for the dehazing urban RS image. The URSHR algorithm combines phase consistency features, multi-scale Retinax theory, and histogram features to restore haze-free images. This algorithm is a feasible and effective method for haze removal of urban RS images and has a good application and promotion value. Chaudhry et al. [43] proposed a framework for image restoration and haze removal. It uses hybrid median filtering and accelerated local Laplacian filtering to dehaze the image and has achieved good results on outdoor RGB images and RS images.

## 4. Physical Dehazing

As discussed in Section 2, the physical dehazing technique is based on the well-known ASM and imposes one or more prior knowledge [44,45] or assumptions on it to reduce the uncertainty of haze removal [46,47].

### 4.1. Dark Channel Prior

He et al. [48] observed a large number of outdoor haze-free images and found that in most of the non-sky patches, at least one color channel has some pixels whose intensity are very low and close to zero. For an arbitrary image J, its dark channel [49,50] $J^{dark}$ is given by:

(18) $$J^{dark}\left(x\right)=\underset{y\in \Omega \left(x\right)}{min}\left(\underset{c\epsilon \left\{R,G,B\right\}}{min}J^{c}\left(y\right)\right),$$

where $J^{c}$ is a color channel of J, and Ω(x) is a local patch centered at x. If J is an outdoor haze-free image, then the value of $J^{dark}$ should be very low or close to zero. Please note that the low intensity in the dark channel is mainly due to shadows of scene, dark objects, and colorful objects or surfaces.

#### 4.1.1. Estimating the Transmission

Equation (18) can be normalized by:

(19) $$\frac{I^{c}\left(x\right)}{A^{c}}=t\left(x\right)\cdot \frac{J^{c}\left(x\right)}{A^{c}}+1-t\left(x\right),$$

Assuming that the value of A is known and the transmission in a local patch Ω(x) is constant, which is defined as $\tilde{t}\left(x\right)$. Then, the minimization operation of Equation (19) is:

(20) $$\underset{y\in \Omega \left(x\right)}{min}\left(\underset{c}{min}\frac{I^{c}\left(y\right)}{A^{c}}\right)=\tilde{t}\left(x\right)\cdot \underset{y\in \Omega \left(x\right)}{\min}\left(\underset{c}{min}\frac{J^{c}\left(y\right)}{A^{c}}\right)+1-\tilde{t}\left(x\right),$$

By imposing DCP into Equation (20), we have:

(21) $$J^{dark}\left(x\right)=\underset{y\in \Omega \left(x\right)}{min}\left(\underset{c\epsilon \left\{R,G,B\right\}}{min}J^{c}\left(y\right)\right)\to 0,$$

Putting Equation (21) into Equation (20), the estimated transmission is simplified as:

(22) $$\tilde{t}\left(x\right)=1-\underset{y\in \Omega \left(x\right)}{min}\left(\underset{c}{min}\frac{I^{c}\left(y\right)}{A^{c}}\right),\;$$

In practice, even on clear days the atmosphere is not absolutely free of any particle. Therefore, the haze still exists when we look at distant objects. Moreover, the presence of haze is a fundamental cue for humans to perceive depth [51,52]. Therefore, it is necessary to retain a certain degree of haze to obtain a better visual effect. It can be modified by introducing a factor ω between [0, 1] in Equation (22), usually setting it to be 0.95, and then Equation (22) is modified as:

(23) $$\tilde{t}\left(x\right)=1-\omega \underset{y\in \Omega \left(x\right)}{min}\left(\underset{c}{min}\frac{I^{c}\left(y\right)}{A^{c}}\right),\;$$

#### 4.1.2. Estimating the Atmospheric Light

To estimate the atmospheric light, He firstly picked the top 0.1% brightest pixels in the dark channel and then recorded the coordinates of these pixels. Finally, the max value of corresponding pixel in the original image is regarded as atmospheric light [48].

#### 4.1.3. Recovering the Scene Radiance

Putting the estimated values of atmospheric light A and transmission t into Equation (11), the haze-free can be obtained by:

(24) $$J\left(x\right)=\frac{I\left(x\right)-A}{t\left(x\right)}+A,$$

The direct attenuation term J(x)t(x) will be very close to zero when the transmission t is close to zero. Therefore, setting a lower bound value $t_{0}$ for transmission. The final scene radiance J is recovered by:

(25) $$J\left(x\right)=\frac{I\left(x\right)-A}{max\left(t\left(x\right),t_{0}\right)}+A.$$

Due to the fact that transmission is not always constant in a patch, the restored image will have block artifacts using a rough transmission. He et al. proposed a soft matting algorithm to optimize the transmission. However, it takes a long time to calculate. Later, He et al. [53] used guided filtering to replace the soft matting. The complexity was reduced, and the computational efficiency was greatly improved. The restored image by DCP has a promising visual result. However, if the target scene is similar to atmospheric light, such as snow, white walls, and sea, satisfactory results will not be obtained.

### 4.2. Non-Local Image Dehazing

According to the fact that a nature image usually contains a lot of repeated colors, Berman et al. [54] develop a non-local dehazing technique, which is different from the patch-wise and pixel-wise dehazing ones. The core idea is to adopt K-means [55] to cluster the image input into 500 haze-line, and then estimate the transmission map using these haze-lines [56]. Having this estimated parameter, a haze-free result can be recovered from single hazy images.

#### 4.2.1. Haze-Lines Clustering

Firstly, $I_{A}$ was defined by the following equation:

(26) $$I_{A}\left(x\right)=I\left(x\right)-A,$$

where the 3D RGB coordinate system is translated such that the air light is at the origin. Combining Equation (11), we can get:

(27) $$I_{A}\left(x\right)=t\left(x\right)\cdot \left[J\left(x\right)-A\right],$$

Then, redefining $I_{A}\left(x\right)$ using spherical coordinates, i.e.,

(28) $$I_{A}\left(x\right)=\left[\gamma \left(x\right),\theta \left(x\right),\varphi \left(x\right)\right],$$

where γ is the distance to the origin, and θ and φ are the longitude and latitude, respectively. It can be noticed from Equation (27) that scene points at different distances differ only in the transmission value. In other words, pixels x and y have similar RGB values in the underlying haze-free image if their [φ,θ ] are similar:

(29) J(x)≈J(y)→{φ(x)≈φ(y),θ(x)≈θ(y)},    ∀t,

Therefore, pixels belong to the same haze-line if their [φ(x),θ(x) ] values are similar.

#### 4.2.2. Estimating Transmission

For a given haze-line defined by J and A, r(x) depends on the object distance:

(30) r(x)=t(x)·‖J(x)−A‖,   0≤t(x)≤1,

Thus, t=1 corresponds to the largest radial coordinate:

(31) $$r_{max}\overset{\mathrm{def}}{=}\|J-A\|,$$

Combining Equations (30) and (31), the estimated transmission can be simplified as:

(32) $$\tilde{t}\left(x\right)=r\left(x\right)/{\hat{r}}_{max},$$

where ${\hat{r}}_{max}\left(x\right)=\underset{x\in H}{\max}\left\{r\left(x\right)\right\}$, H is a haze-line containing the haze-free pixel. After regularization for $\tilde{t}$, the dehazed image can be obtained by ASM. This method can deal with most hazy examples well. However, it may perform poorly for some hazy images that do not satisfy the introduced priors.

### 4.3. Other Physical Dehazing Methods

TAN [57] observed that haze-free images have higher contrast compared with the hazy images, and maximized the contrast per patch, while maintaining a global coherent image. This algorithm enhances the contrast of the image and improves its visibility. Unfortunately, color oversaturation and halo effect are visible in the images after dehazing. Fattal [58] firstly assumed that the albedo of the local image regions is a constant, and the transmission and surface shading are locally uncorrelated. Then, the independent component analysis (LCA) is used to estimate the albedo. As expected, the performance of this method mainly depends on the statistical characteristics of the input data to a certain extent, thus insufficient color information is bound to lead to unreliable statistical estimates.

### 4.4. Remote Sensing Image Dehazing Using DCP

Since RS images are imaged from a high altitude, they generally do not include the sky area. Wang [59] believes that most areas’ dark channel value is maintained at a relatively low level. Therefore, the blocking phenomenon has little effect on the dehazing RS image. This enables omitting the transmission refinement process, thus simplifying the dehazing process and improving the calculation efficiency. Zheng et al. [60] introduced the failure point based on the DCP. They set the failure point threshold, and effectively avoided the bright objects’ influence on dehazing RS images. Li et al. [61] used the median filter method to refine the transmission and improve aerial images’ calculation efficiency. Wang et al. [62] proposed a block-based DCP method for remotely sensed multispectral images, using the atmospheric light surface hypothesis to replace the global atmospheric light, making RS images better restored. Long et al. [63,64] used a low-pass Gaussian filter to refine the atmospheric veil and redefined the transmission to eliminate color distortion. Dai et al. [65] generated a dark channel image by directly obtaining the minimum of the three channels of each pixel of the RS image.

## 5. Data-Driven Based Dehazing

With the continuous development of deep learning theory, convolution neural network (CNN) [66,67,68,69,70] has been utilized and achieved good results in face recognition, image segmentation, and other fields. Image dehazing, as an issue of great concern in image processing, has also attracted many scholars’ attention. Most data-driven based dehazing techniques have achieved tremendous success compared with the traditional haze removal methods.

### 5.1. DehazeNet

**Figure 3:**
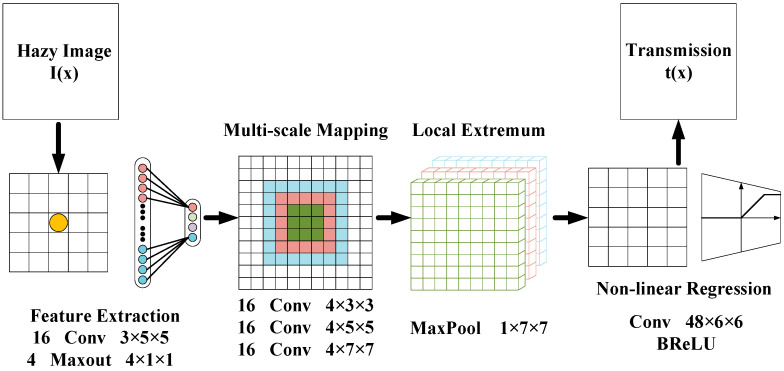
The architecture of DehazeNet.

DehazeNet [71,72] was proposed by Cai et al. [73] in 2016. It uses a multi-level architecture based on a CNN, which takes a hazy image as an input and outputs its transmission map. Then, according to this estimated output, they restored the haze-free image based on the ASM. The structure of DehazeNet is shown in Figure 3.

DehazeNet employs feature extraction, multi-scale mapping, local extremum, and nonlinear regression to calculate the transmission map of a hazy image.

Feature extraction: It consists of a convolutional layer and a Maxout unit [74], which convolves the hazy image with appropriate filters, and then uses nonlinear mapping to obtain the feature map. The Maxout unit is a simple feed-forward nonlinear activation function used in multi-layer perceptron or CNNs. When it is used in CNNs, it generates a new feature map by taking a pixel-wise maximization operation over k affine feature maps.

Multi-scale mapping: It is composed of 16 convolution kernels with sizes of 3 × 3, 5 × 5, and 7 × 7 to adapt to features of different sizes and scales. In previous studies, multi-scale features have been proven to have significant effects on image dehazing.

Local extremum: The neighborhood maximum is considered under each pixel to overcome local sensitivity. In addition, the local extremum is in accordance with the assumption that the medium transmission is locally constant, and it is common to overcome the noise of transmission estimation.

**Figure 4:**
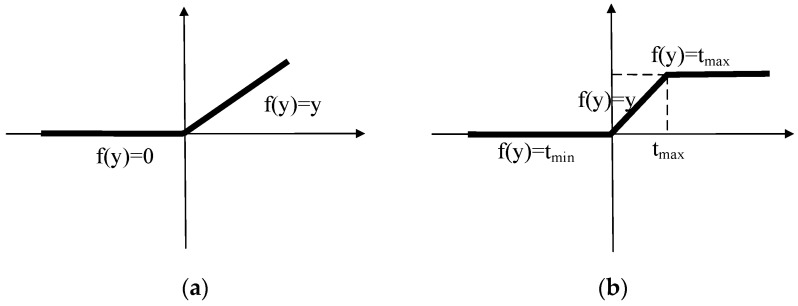
Rectified linear unit (ReLU) and bilateral rectified linear unit (BReLU). (**a**) ReLU. (**b**) BreLU.

Nonlinear regression: Since ReLU [75,76] is only prohibited when the value is less than zero and the output value of the last layer of the image reconstruction task is between 0 and 1, it may cause the overflow. Therefore, the value greater than one is suppressed. To this end, a Bilateral Rectified Linear Unit (BReLU) [77] activation function is proposed by Cai et al. to overcome this limitation (as shown in Figure 4). As a novel linear unit, BReLU maintains bilateral constraints and local linearity.

Experiments show that the system has better performance than existing methods. However, ASM relies on a single light source without considering multi-light source, and the dehazing effect in the distant area needs to be improved.

### 5.2. MSCNN

**Figure 5:**
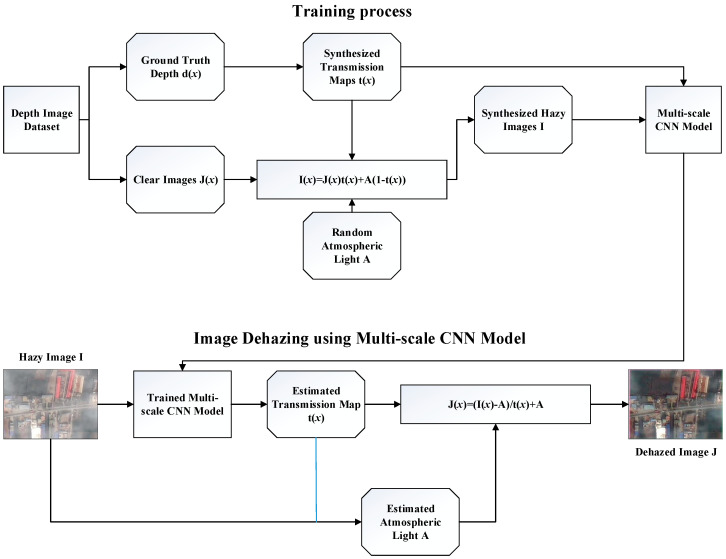
Main steps of the proposed single-image dehazing algorithm.

DehazeNet extracts the feature map through a convolution neural network to get the transmission map, but the transmission obtained through DehazeNet is not refined. Therefore, Ren et al. [78] designed a multi-scale CNN for image dehazing. As shown in Figure 5, the original hazy image is used as input, the transmission map first estimated by a coarse-scale network and then refined by a fine-scale network.

The coarse-scale CNN predicts the scene’s overall transmission map, which is composed of a multi-scale convolution layer, a pooling layer, an up-sampling layer [79,80,81,82], and a linear combination layer. The convolutional layer is designed to have different sizes of convolution kernels to learn multi-scale features. Each convolutional layer is followed by a ReLU layer, a pooling layer, and an upsampling layer. The linear combination layer linearly combines the features of the previous layer to obtain a rough transmission map, which will be used as the input of the fine-scale CNN.

The fine-scale CNN is to refine the transmission map output by the coarse-scale neural network. It is similar to the coarse-scale network. The rough transmission map is input into a fine-scale CNN. They work together to obtain a refined transmission map.

As discussed in [78], the performance of haze-free results using this training network can be improved compared to those of traditional techniques. Despite this, the max-pooling adopted in the model will result in loss of details, and the image dehazing at nighttime is not reliable as well.

### 5.3. AOD-NET

DehazeNet and MSCNN estimate the atmospheric light by DCP. However, the estimated value may cause errors when the color of the object in the hazy image is close to the atmospheric light. Moreover, the separate estimation of transmission and atmospheric light may further increase the error and affect the result. To solve this problem, Li et al. [83] proposed the first end-to-end trainable dehazing model, which can directly restore the haze-free image from the hazy image rather then relying on any intermediate parameter estimation. The AOD-Net [83] model transforms the ASM Equation (11), and it is calculated as:

(33) $$J\left(x\right)=\frac{1}{t\left(x\right)}\cdot I\left(x\right)-A\cdot \frac{1}{t\left(x\right)}+A,$$

The core idea is to combine the transmission t and the atmospheric light value A into K(x), which is calculated as:

J(x)=K(x)·I(x)−K(x)+b, 

where,

(34) $$K\left(x\right)=\frac{\frac{1}{t\left(x\right)}\cdot \left(I\left(x\right)-A\right)+\left(A-b\right)}{I\left(x\right)-1},$$

b is a constant bias, and the default value is 1.

**Figure 6:**
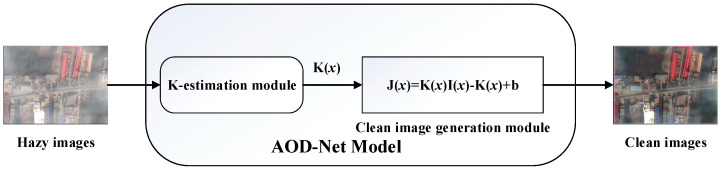
The diagram of AOD-Net.

AOD-Net is composed of two parts: K-estimation module and clean image generation module (as shown in Figure 6). Parameters in K(x) vary with the input hazy image. The model is trained by minimizing the loss between the output image J and the clear image. Continuously reducing the loss, thereby outputting the haze-free image J. This model has greatly improved in terms of PSNR and SSIM. In addition, this end-to-end design can easily embed the model into other data-driven ones, thereby improving the performance of image processing tasks.

### 5.4. FD-GAN

**Figure 7:**
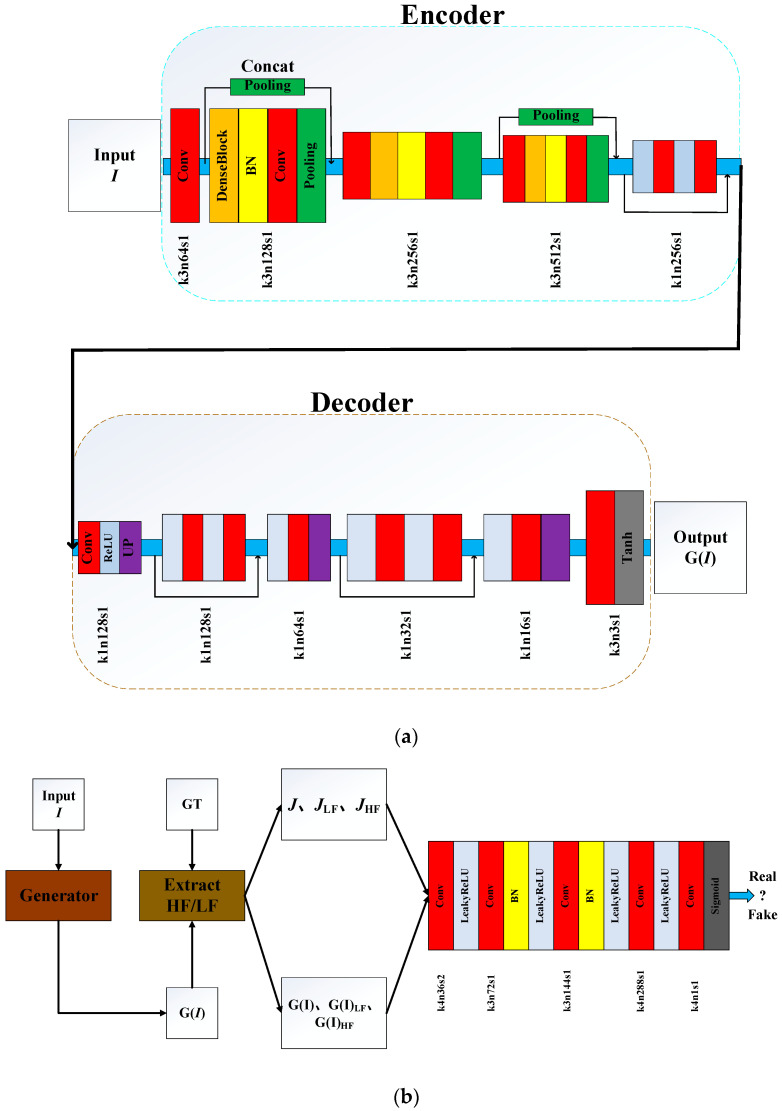
The structure of FD-GAN. (**a**) Generator. (**b**) Fusion-discriminator.

Yu et al. [84] proposed a fully end-to-end Generative Adversarial Network with Fusion-discriminator (FD-GAN) for image dehazing. FD-GAN consists of Generator and Fusion-discriminator (as shown in Figure 7). The Generator including decoder and encoder can directly generate the dehazed images G(I) without estimation of parameters. The encoder contains three dense blocks, including a series of convolutional, batch normalization (BN), and ReLU layers. The decoder uses the nearest-neighbor interpolation for up-sampling to recover the size of feature maps to the original resolution gradually. The low-frequency (LF) component and high-frequency (HF) component were obtained by Gaussian filter and Laplace operator, respectively. Yu et al. concatenate the G(I) (or Ground truth image J) and its corresponding LF and HF as a sample, then feed it into the Fusion-discriminator. The LF and HF can assist the discriminator to distinguish the differences between hazy and ground truth images well, and can guide the generator to output more natural and realistic hazy-free images.

### 5.5. Remote Sensing Image Dehazing Using Data-Driven

Guo et al. [85] proposed an end-to-end RSDehazeNet for haze removal. Guo et al. utilize both local and global residual learning strategies in RSDehazeNet for fast convergence with superior performance. To obtain enough RS images for CNN training, Guo et al. proposed a novel haze synthesis method to generate realistic hazy multispectral images by modeling the wavelength-dependent and spatial-varying characteristics of haze in RS images. Jiang et al. [86] proposed a multi-scale residual convolutional neural network (MRCNN) for haze removal of RS images. MRCNN uses three-dimensional convolution kernels to extract spatial-spectral correlation information and abstract features from the surrounding neighborhoods for haze transmission estimation, achieving extremely low verification error and test error. Qin et al. [87] proposed a novel dehazing method based on a deep CNN with the residual structure for multispectral RS images. First, connect CNN individuals with multiple residual structures in parallel, and each individual is used to learn the regression from a hazy image to a clear image. Then, the individual output of CNN is fused with the weight map to produce the final dehazing result. This method can accurately remove the haze in each band of multispectral images under different scenes. Chen et al. [88] proposed an end-to-end hybrid high-resolution learning network framework termed H2RL-Net to remove a single satellite image haze. It can deliver significant improvements in RS image owing to its novel feature extraction architecture. Mehta et al. [89] proposed SkyGAN for haze removal in aerial images, including a hazy-to-hyperspectral (H2H) module, and a conditional GAN (cGAN) module for dehazing. A high-quality result can be produced when evaluating this algorithm on the SateHaze1k dataset and the HAI dataset. Huang et al. [90] proposed the self-supporting dehazing network (SSDN) to improve the efficiencies in the restoration of content and details. The SSDN introduced the self-filtering block to raise the representation abilities of learned features and achieved good performance.

## 6. Remote Sensing Dehazing Image Quality Evaluation

After realizing the RS image haze removal according to the aforementioned algorithms, it is also crucial to use some quality metrics to evaluate the image quality. This section first introduces several commonly used metrics in detail and then uses them to assess the result dehazed by different methods.

### 6.1. Mean Squared Error (MSE)

The mean squared error (MSE) is a metric used to estimate the error between the actual image and the restored image, which is computed as [91,92]:

(35) $$\mathrm{MSE}=\frac{1}{P\cdot Q}\sum_{x=1}^{P}\sum_{y=1}^{Q}{\left(f\left(x,y\right)-h\left(x,y\right)\right)}^{2},\;$$

where f(x,y) and h(x,y) represent the real image and the restored image, respectively. P and Q represent the length and width of the image, and x and y are the coordinate of the pixel in an image.

### 6.2. Mean Absolute Error (MAS)

The mean absolute error (MAE) represents the mean of the absolute error between the predicted and the observed. Compared to MSE, it can avoid the problem of errors cancelling each other out and basically provides a positive integer ranging from 0 to 255 for an 8-bit image. Formally, it is computed by:

(36) $$\mathrm{MAE}=\frac{1}{P\cdot Q}\sum_{x=1}^{p}\sum_{y=1}^{Q}\left|f\left(x,y\right)-h\left(x,y\right)\right|,$$

### 6.3. Peak Signal-to-Noise Ratio (PSNR)

PSNR is the most common and widely used objective metric for ranking the quality of images. It evaluates the ratio of actual pixels value and the evaluated error using MSE. It can be computed by [91,92]:

(37) $$\mathrm{PSNR}=10log_{10}\frac{M^{2}P\cdot Q}{\sum_{x=1}^{P}\sum_{y=1}^{Q}{\left(f\left(x,y\right)-h\left(x,y\right)\right)}^{2}}=10log_{10}\frac{M^{2}}{MSE},$$

where M is the image gray level, generally taking 255, and n is the binary digit used by a pixel, generally 8-bits.

### 6.4. Structural Similarity Index (SSIM)

SSIM is a metric used to measure the similarity of pictures and can also be used to judge the quality of pictures after compression [93]. In general, a larger SSIM value means a smaller image distortion. Natural images are extremely structural and reflect the correlation among pixels. It carries essential information about the structure of the object in the visual scene, and is computed as [92]:

(38) $$\mathrm{SSIM}=\frac{\left(2\mu_{x}\mu_{y}+c_{1}\right)\cdot \left(2\sigma_{xy}+c_{2}\right)}{\left(\mu_{x}^{2}+\mu_{y}^{2}+c_{1}\right)\cdot \left(\sigma_{x}^{2}+\sigma_{y}^{2}+c_{2}\right)},$$

where $\mu_{x}$, $\mu_{y}$ and $\sigma_{x}^{2}$, $\sigma_{y}^{2}$ are the mean and variance of x and y, respectively,$\;c_{1}={\left(r_{1}T\right)}^{2}$, $c_{2}={\left(r_{2}T\right)}^{2}$ is a constant used to maintain stability, $r_{1}=0.01$, $r_{2}=0.03$, $\sigma_{xy}$ is the covariance of x and y, and T is the dynamic range of the pixel value, generally T=255.

### 6.5. Quantitative Comparison

**Figure 8:**
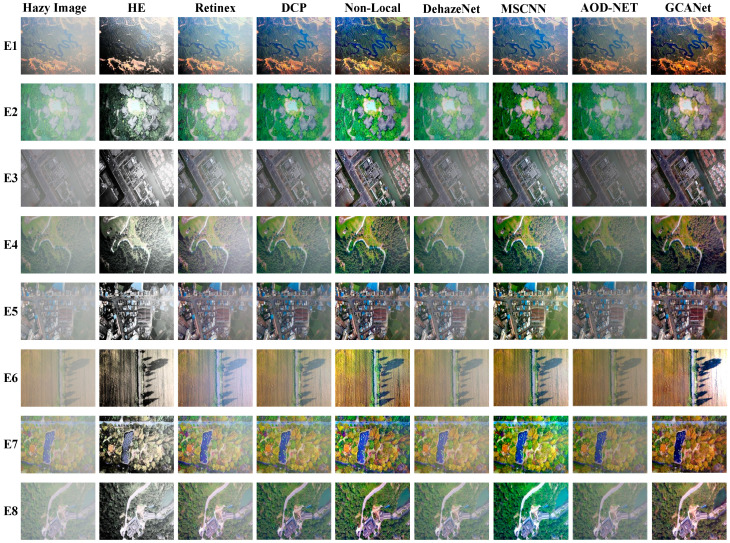
Comparison of RS image dehazing methods discussed above.

To check the recovery performances of different techniques, the above mentioned methods (including HE, Retinex, DCP, Non-Local, DehazeNet, MSCNN, AOD-NET, and GCANet [94]) were tested on eight challenging real-world RS hazy pictures. The selected RS images and the results dehazed by the compared approaches are shown in Figure 8. It can be seen from this figure that RS images dehazed by traditional enhancement methods, i.e., HE and Retinex, have high contrast, while they lose some details, e.g., the brighter area in the upper right corner of E1 and the darker area on the left in E2. Moreover, the results of the physical dehazing, i.e., DCP and Non-Local, may lead to some darker RS outputs than they should be (see the DCP result of E5). In contrast, despite the fact that data-driven dehazing is able to produce a high-quality haze-free scene for most given examples, they may fail to the case with heavy haze.

**Figure 9:**
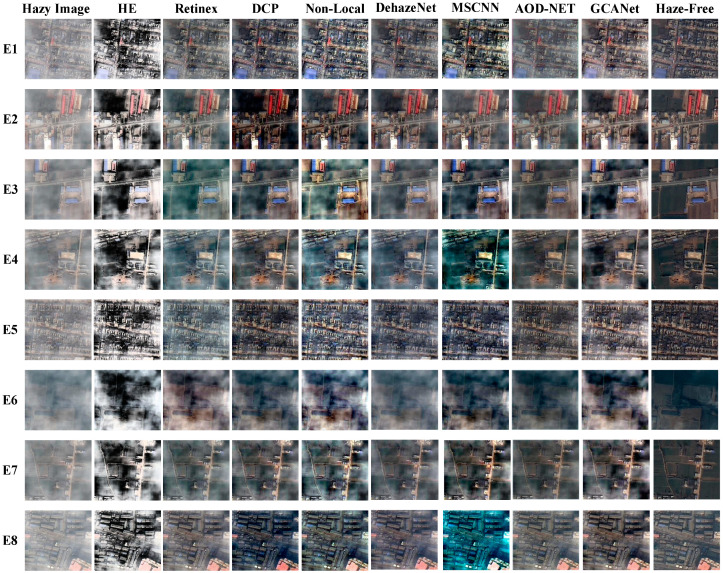
Comparison of RS image dehazing methods discussed above.

To accurately rank the performance of above compared techniques, we also tested them on eight simulated RS data consisting of hazy image and ground truth. The corresponding recovery results are shown in Figure 9. As expected, the results on simulated input also confirm that both image enhancement, physical model, and data-driven have a somewhat ability to remove the haze cover in an image, i.e., having a good output on a special example. However, they do not work well on the images with various scenes.

Furthermore, we employ MSE, MAE, PSNR, and SSIM to access the restoration quality of selected dehazing methods, as summarized in Table 1. It can be found that data-driven dehazing has more potential to achieve RS image dehazing since it roughly wins the best score in terms of all used evaluation index.

## 7. Remote Sensing Technology Application

### 7.1. Monitoring and Control of Geological Disaster

Geological disasters, such as landslides, mudslides, and ground fissures, seriously endanger human life and wealth security. A high-quality RS image can help us roughly investigate the overall damage in the disaster area. However, RS data may lose its value when it is obscured by clouds and haze. Therefore, removing the haze from hazy RS images is very significant in geological disaster monitoring and control.

### 7.2. Urban Planning

The main task of urban planning is to obtain comprehensive urban spatial information. Using RS technology to take city images can easily and accurately capture such information, but there are many factories and construction sites located in cities, which result in a large number of smoke over cities, and thus blurs the RS images. After dehazing the RS data, comprehensive planning and development of the city can be carried out reliably.

### 7.3. Military Application

It is well-known that valuable military intelligence can be obtained from clear RS images, which can be used to discover missiles, identify troops, confirm airports, monitor changes in forces, and make operation plans. Due to the haze interference, the data collected in military will also have the characteristics of low contrast and dim colors. Therefore, the haze removal technique can be useful to handle this issue.

## 8. Future Efforts

Researchers have done a large amount of research work on RS image dehazing and have achieved a promising result for most cases. However, there is still a lot of vital work to be further studied.

### 8.1. Drawback of ASM

The image dehazed by ASM will have a dim effect since ASM fails to consider the light trapping phenomenon related to the texture density and scene depth. In other words, ASM considers that all scenes in the image are directly illuminated by the atmospheric light, while ignoring the influence of uneven illumination. To address the above problems, many useful methods [18,23] which optimize the robustness of the ASM are proposed. Although the dim effect is solved to a certain extent, uneven haze remains a challenge. Therefore, it is a challenging problem to use a more robust physical model to describe complex scenes.

### 8.2. Priori Limitation

Most of the current existing methods are based on ASM, and achieve haze removal using latent prior on ASM. However, due to the fact that prior cannot fully satisfy all images or data, it is difficult to ensure the recovery performance of these approaches. In some conditions, especially for an image with heavy haze, haze removal using prior will be ineffective. Benefitting from the learning mechanism, building a deep architecture or Bayesian framework to integrate the remarkable merit of each algorithm is a good choice, so that a better haze-free result can be obtained.

### 8.3. Real-Time Dehazing

Although current dehazing algorithms are able to effectively remove haze for a single image, most of them still have a common problem, i.e., lacking real-time performance. This means that these dehazing methods still cannot support the normal operation of computer vision systems that need high efficiency processing. In a word, a “good” haze removal algorithm must have reliable recovery capability and low computational complexity simultaneously. To the best of our knowledge, all the existing algorithms reduce the complexity by optimizing the algorithm itself. In fact, using hardware (graphics processing unit) to accelerate the processing may be more effective than the previous work.

### 8.4. Drawback of Data-Driven

On the one hand, the data-driven restoration quality depends on the selection of the training dataset. However, almost all open datasets are artificially synthesized rather than being collected from the real-world, especially for RS data. This is bound to lower the dehazing effect on real-world RS data. On the other hand, data-driven dehazing is similar to a “black box”, which lacks interpretability and is specifically theoretical despite its effectiveness. Therefore, researchers could combine statistical learning with symbolic computation and construct an uneven haze image dataset to obtain more natural and realistic hazy-free images.

## 9. Conclusions

In conclusion, this paper details the degradation mechanism of hazy data and the corresponding physical model, i.e., ASM. Then, a brief introduction of RS images and attributes of each type of dehazing algorithm were discussed categorically. In short, image enhancement neglects the imaging theory of hazy data and only stresses the enhancement of local or global contrast as much as possible. In addition, physical dehazing extracts the parameters by imposing latent prior knowledge on ASM, thereby it can restore a haze-free scene from the hazy image physically. Moreover, data-driven dehazing makes use of the powerful learning ability of neural network to find the mapping relationship between hazy data and the corresponding haze-free one or transmission map. Therefore, its success on dehazing performance mainly lies in the training dataset used to drive the expected models. Finally, the commonly used quantitative metrics and the application scenario of RS dehazing approaches were also illustrated. Furthermore, we emphasized some challenging problems faced by these RS dehazing methods that enlighten the future efforts in this topic.

## Figures and Tables

**Table 1:** Comparison of evaluation metrics on images shown in Figure 7.

Metric	Image	HE	Retinex	DCP	Non-Local	DehazeNet	MSCNN	AOD-NET	GCANet
MSE	E1	58.0250	65.3694	40.1377	63.0135	45.5643	41.8578	45.9550	60.8299
	E2	58.4546	65.3156	2.3213	18.8858	30.1598	58.2286	48.4368	45.0116
	E3	63.8380	74.9954	58.0916	70.6542	78.5197	50.8604	54.8527	61.5088
	E4	65.9776	83.2367	75.1314	74.8605	79.7631	38.7678	58.7273	66.4670
	E5	66.5203	84.4062	50.1036	78.2738	68.5316	57.0354	57.3905	79.1826
	E6	66.4734	78.6077	74.9392	76.3182	84.3603	60.7811	58.9837	65.4599
	E7	64.6617	79.2677	71.7706	68.9340	73.9023	50.3411	58.6004	60.0329
	E8	52.5054	50.0531	5.2568	16.8775	29.1833	28.4264	40.3536	21.0587
MAE	E1	16.8697	9.3487	5.0937	10.2678	5.4562	7.0095	6.0234	9.6560
	E2	14.8499	9.2820	0.3630	2.1677	3.2229	7.1188	6.2519	5.4573
	E3	20.9289	13.0207	8.6793	16.4741	12.8597	8.0818	7.4172	11.7235
	E4	23.5596	18.7855	13.2110	16.1918	14.5504	6.8617	8.2913	11.2540
	E5	21.3970	19.7671	6.4796	15.3561	9.9177	8.3746	7.8404	16.3443
	E6	25.9595	21.0835	12.8393	21.4060	18.4271	9.6946	7.9932	16.2544
	E7	24.5050	14.9594	12.4878	14.3627	11.9880	8.8039	8.1327	11.5565
	E8	14.0012	6.1392	0.6912	1.8448	3.0357	4.6955	4.8347	2.2535
PSNR	E1	11.3018	17.3161	19.9218	16.1124	20.5306	16.7291	19.1775	16.4637
	E2	12.4495	17.3834	16.0288	21.2499	22.6673	19.2980	19.1143	20.1044
	E3	10.0178	15.0239	17.2843	12.5169	15.3483	16.9267	18.1787	14.5837
	E4	9.1166	12.3160	14.7143	12.9381	14.2402	16.4077	17.4782	15.5258
	E5	9.9918	12.0322	19.2096	13.7839	16.9787	17.3780	17.9371	13.0886
	E6	8.2387	10.8556	14.9927	10.4802	12.7805	16.1591	17.7590	12.0327
	E7	8.6274	14.0277	14.9921	13.4883	15.6290	15.9782	17.6301	14.5927
	E8	12.1672	19.9542	17.5611	21.0496	22.7793	15.2877	20.5717	21.1517
SSIM	E1	0.6244	0.8533	0.8687	0.7670	0.8870	0.7742	0.8429	0.8416
	E2	0.6290	0.8297	0.6681	0.8649	0.8900	0.8723	0.8373	0.8581
	E3	0.5179	0.6285	0.7921	0.6042	0.7909	0.7427	0.8061	0.7321
	E4	0.4934	0.6150	0.7554	0.6082	0.6434	0.5457	0.7807	0.7662
	E5	0.6196	0.6911	0.8652	0.7251	0.7991	0.8230	0.8231	0.7827
	E6	0.3996	0.3357	0.7244	0.5104	0.6864	0.7246	0.7937	0.4873
	E7	0.4193	0.7345	0.7478	0.6231	0.7745	0.7045	0.7783	0.6874
	E8	0.5479	0.8689	0.7142	0.8267	0.8776	0.3633	0.8445	0.8437

## Data Availability

Not applicable.
